# Realizing a High‐Performance Na‐Storage Cathode by Tailoring Ultrasmall Na_2_FePO_4_F Nanoparticles with Facilitated Reaction Kinetics

**DOI:** 10.1002/advs.201900649

**Published:** 2019-05-07

**Authors:** Fanfan Wang, Ning Zhang, Xudong Zhao, Lixuan Wang, Jian Zhang, Tianshi Wang, Fanfan Liu, Yongchang Liu, Li‐Zhen Fan

**Affiliations:** ^1^ Beijing Advanced Innovation Center for Materials Genome Engineering Institute for Advanced Materials and Technology University of Science and Technology Beijing Beijing 100083 China; ^2^ College of Chemistry and Environmental Science Hebei University Baoding 071002 China; ^3^ Key Laboratory of Advanced Energy Materials Chemistry (Ministry of Education) Nankai University Tianjin 300071 China; ^4^ School of Electrical Engineering and Automation Tianjin Polytechnic University Tianjin 300387 China

**Keywords:** binder‐free cathodes, carbon nanofibers, reaction kinetics and mechanism, sodium‐ion batteries, ultrasmall Na_2_FePO_4_F nanoparticles

## Abstract

In this paper, the synthesis of ultrasmall Na_2_FePO_4_F nanoparticles (≈3.8 nm) delicately embedded in porous N‐doped carbon nanofibers (denoted as Na_2_FePO_4_F@C) by electrospinning is reported. The as‐prepared Na_2_FePO_4_F@C fiber film tightly adherent on aluminum foil features great flexibility and is directly used as binder‐free cathode for sodium‐ion batteries, exhibiting admirable electrochemical performance with high reversible capacity (117.8 mAh g^−1^ at 0.1 C), outstanding rate capability (46.4 mAh g^−1^ at 20 C), and unprecedentedly high cyclic stability (85% capacity retention after 2000 cycles). The reaction kinetics and mechanism are explored by a combination study of cyclic voltammetry, ex situ structure/valence analyses, and first‐principles computations, revealing the highly reversible phase transformation of Na_2_Fe^II^PO_4_F ↔ NaFe^III^PO_4_F, the facilitated Na^+^ diffusion dynamics with low energy barriers, and the desirable pseudocapacitive behavior for fast charge storage. Pouch‐type Na‐ion full batteries are also assembled employing the Na_2_FePO_4_F@C nanofibers cathode and the carbon nanofibers anode, demonstrating a promising energy density of 135.8 Wh kg^−1^ and a high capacity retention of 84.5% over 200 cycles. The distinctive network architecture of ultrafine active materials encapsulated into interlinked carbon nanofibers offers an ideal platform for enhancing the electrochemical reactivity, electronic/ionic transmittability, and structural stability of Na‐storage electrodes.

## 1 Introduction

Sodium‐ion batteries (SIBs) have aroused tremendous attention and emerged as the promising candidate for large‐scale electric energy storage owing to their advantages of abundant Na resources, low cost, feasibility of using aluminum as both cathode and anode current collectors, and similar working principle to that of lithium‐ion batteries (LIBs).^1^, ^2^, ^3^, ^4^ Nevertheless, inert reaction kinetics and low power/energy density resulted from the relatively large radius and heavy mass of Na^+^ ion are the major obstacles that hinder the widespread applications of SIBs.^5^, ^6^, ^7^ Therefore, it is imperative to develop reliable Na‐storage electrodes with superior rate capability and long‐term cycle life,^8^, ^9^, ^10^ especially cathode materials which are more difficult for sodium‐ion transport.^11^, ^12^, ^13^ In research to date, there are four main types of cathode materials suitable for SIBs, namely, transition metal oxides,^14^, ^15^, ^16^ polyanionic compounds,^17^, ^18^, ^19^, ^20^ Prussian blue analogues,^21^, ^22^, ^23^ and organic sodium salts.^24^, ^25^ Among them, the layered iron‐based fluorophosphate of Na_2_FePO_4_F is particularly attractive by virtue of its facile 2D Na^+^ pathways formed by interconnected PO_4_ tetrahedra and FeO_4_F_2_ octahedra, great structural stability with small volume variation (only 3.7%) during sodiation/desodiation, huge availability of iron ore reserves, satisfactory theoretical capacity (124 mAh g^−1^), and decent operating voltage (≈3.0 V vs Na^+^/Na) induced by polyanion effect.^26^, ^27^, ^28^ Unfortunately, the primary two challenges facing Na_2_FePO_4_F lie in the low electronic conductivity correlated with poor rate property and the undesirable actual capacity caused by inferior active materials utilization (especially for bulk materials).^29^ Hence, it is urgently necessary to boost the reaction kinetics and activity of Na_2_FePO_4_F cathode toward improved electrochemical performance.

At present, considerable efforts have been devoted to fabricating the carbon‐coated nanostructured Na_2_FePO_4_F aiming at solving the abovementioned issues. In 2007, Nazar's group first proposed that the Na_2_FePO_4_F nanoparticles (≈200 nm) with a carbon coating layer of 2–4 nm were electrochemically active in SIBs.^30^ Thereafter, a Na‐storage capacity of around 100 mAh g^−1^ was obtained for nano‐Na_2_FePO_4_F by Tarascon and co‐workers.^31^ Wang's group then synthesized the carbon‐coated porous hollow Na_2_FePO_4_F spheres (diameter of 500 nm, wall thickness of 80 nm) and extended the cycle life to 750 cycles with a capacity retention of 80%.^32^ Dunn and co‐workers further reduced the size of Na_2_FePO_4_F nanoparicles (enwrapped by graphene) to 15–25 nm, leading to rapid Na^+^ diffusion kinetics and thus a respectable rate capability of 60 mAh g^−1^ even at 10 C.^27^ Through very encouraging findings, more decreased particle size (<5 nm) and more uniform carbon coating with regard to this material are highly anticipated to simultaneously realize the high‐rate performance and long lifespan. On the other hand, although Liu's and Yang's teams pioneeringly probed the structural evolution of Na_2_FePO_4_F upon Na‐ion insertion/extraction using in operando X‐ray diffraction (XRD), and discovered a single‐electron phase transition of Na_2_FePO_4_F ↔ NaFePO_4_F with the intermediate phase of Na_1.5_FePO_4_F,^28^, ^33^ the thorough understanding of Na^+^ (de)intercalation sites and migration paths in the Na_2_FePO_4_F crystal, as well as the relevant reaction dynamics, has not yet been well established.

Herein, ultrasmall Na_2_FePO_4_F nanoparticles (≈3.8 nm) homogeneously embedded in porous nitrogen‐doped carbon nanofibers (labeled as Na_2_FePO_4_F@C) are successfully prepared through electrospinning and subsequent annealing processes. To the best of our knowledge, this is the first report on such a distinctive nanostructure tailored for Na_2_FePO_4_F. The as‐prepared Na_2_FePO_4_F@C fiber membrane is firmly adhered to aluminum foil and directly used as a binder‐free cathode for SIBs, enabling both high rate performance (117.8 mAh g^−1^ at 0.1 C in comparison of 46.4 mAh g^−1^ at 20 C) and unprecedentedly high cycling stability (85% capacity retention over 2000 cycles). By means of electrochemical analyses, ex situ structure/valence detections, and first‐principles computations, the highly reversible phase transformation, and the facilitated reaction kinetics concerning fast ionic diffusion, proper pseudocapacitance contribution, and low activation energy of the Na_2_FePO_4_F@C cathode are demonstrated. In addition, soft package Na‐ion full batteries are rationally constructed by pairing the Na_2_FePO_4_F@C nanofibers cathode with the pure carbon nanofibers anode, showing great application prospects with high energy density and long cycle life.

## 2 Results and Discussion

**Figure 1:**
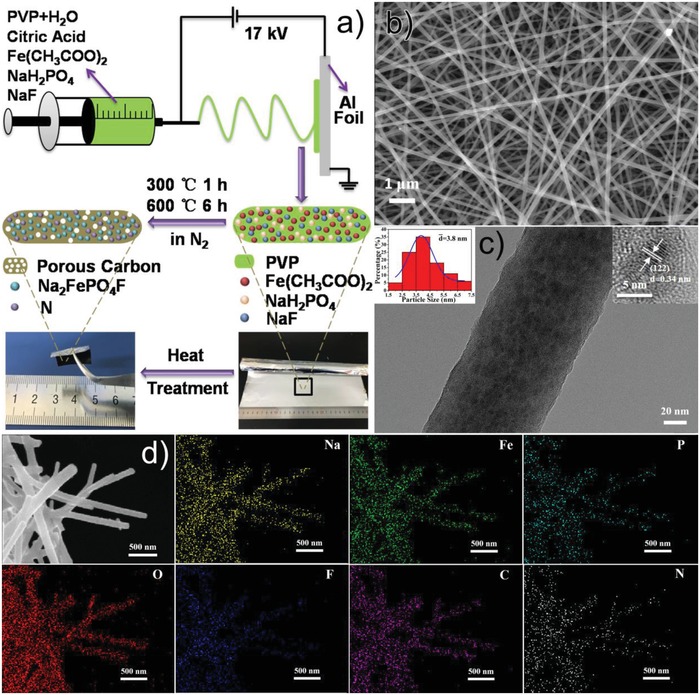
a) Schematic fabrication process of the Na_2_FePO_4_F@C nanofibers. b) SEM, c) TEM (insets are the Na_2_FePO_4_F particle size distribution diagram and HRTEM image), and d) SEM EDS element mapping images of the as‐synthesized Na_2_FePO_4_F@C.

**Figure** **1**a schematically illustrates the preparation process for the Na_2_FePO_4_F@C nanofibers. Briefly, an aqueous solution of polyvinylpyrrolidone (PVP), Fe(CH_3_COO)_2_, NaH_2_PO_4_, and NaF was first electrospun to be smooth and continuous nanofibers (Figure S1, Supporting Information) adherent on aluminum foil. Citric acid was used to protect the Fe^2+^ from being oxidized. Thereafter, the as‐spun fibers underwent a thermal treatment in N_2_ atmosphere during which PVP was carbonized and the metal salts decomposed and then chemically combined to form the Na_2_FePO_4_F. It is worth noticing that porosity due to the released gases and nitrogen‐doping owing to the N‐rich character of PVP were introduced to the generated carbon nanofibers during the calcination process.^34^ The obtained Na_2_FePO_4_F@C nanofibers weaved into a flexible membrane tightly attached onto Al foil, which can be directly adopted as binder‐free cathode for SIBs without the mechanical milling or slurry casting procedure in traditional battery processing. Figure 1b shows the scanning electron microscopy (SEM) image of the as‐prepared Na_2_FePO_4_F@C, revealing its uniform nanofiber morphology with an average diameter of 130 nm. Transmission electron microscopy (TEM) image in Figure 1c further indicates that all of the ultrasmall Na_2_FePO_4_F nanograins (mean size of 3.8 nm) are homogeneously embedded in the carbon nanofibers (high‐magnification TEM image is provided in Figure S2, Supporting Information), the clear lattice fringes with a *d*‐spacing of 0.34 nm in the high‐resolution TEM (HRTEM) image can be well assigned to the (122) plane of orthorhombic Na_2_FePO_4_F. As far as we know, the achieved Na_2_FePO_4_F nanoparticles are the smallest ones ever reported. Figure 1d displays the SEM energy‐dispersive spectrometer (EDS) mapping images of the Na_2_FePO_4_F@C nanofibers, demonstrating uniform distributions of Na, Fe, P, O, F, C, and N elements along the fibrous outline.

**Figure 2:**
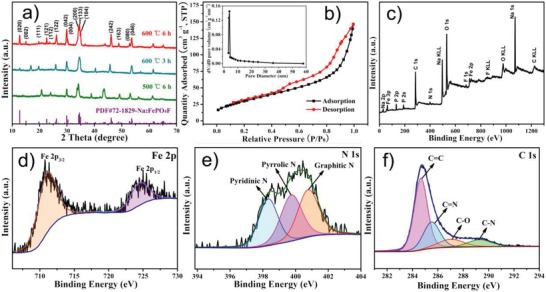
a) XRD patterns of the samples under different annealing conditions. b) N_2_ adsorption–desorption isotherm of the Na_2_FePO_4_F@C nanofibers (inset is the pore size distribution curve). c) Survey XPS spectrum, and d–f) high‐resolution Fe 2p, N 1s, and C 1s XPS spectra of the Na_2_FePO_4_F@C.

Indeed, the calcination temperature and time play crucial roles in building the ideal Na_2_FePO_4_F@C morphology and microstructure. As shown in **Figure** **2**a, the sample annealed at 600 °C for 6 h features XRD peaks in great coincidence with the standard orthorhombic Na_2_FePO_4_F structure (Pbcn space group, JCPDS No. 72‐1829).^27^, ^30^, ^31^, ^32^, ^35^ The Le Bail refinement of the XRD pattern (Figure S3, Supporting Information) indicates that the cell parameters are *a* = 5.2354 Å, *b* = 13.8365 Å, *c* = 11.7734 Å, and *V* = 852.858 Å^3^, respectively, also in good accordance with the previous reports regarding Na_2_FePO_4_F.^29^, ^30^, ^31^ However, the target phase cannot be well formed under a low temperature (500 °C) or a short heating time (3 h), a high temperature (700 °C) or a long sintering time (9 h) results in larger Na_2_FePO_4_F nanoparticles that are not totally confined in the carbon nanofibers (Figures S4 and S5, Supporting Information). Figure 2b presents the N_2_ adsorption–desorption isotherm of Na_2_FePO_4_F@C, the typical type‐IV behavior with an obvious hysteresis loop ranging from 0.4 to 1.0 (*P*/*P*_0_) suggests the existence of mesopores among the nanofibers,^34^, ^36^ and the pore diameters are mainly distributed around 4.2 nm based on the Barrett–Joyner–Halenda (BJH) model. Moreover, the specific surface area is measured to be 112.3 m^2^ g^−1^ according to the Brunauer–Emmett–Teller (BET) method. To gain insight into the chemical content and valence of Na_2_FePO_4_F@C, X‐ray photoelectron spectroscopy (XPS) was performed. From the survey spectrum (Figure 2c), no impurity signals are detected, and the atomic ratio of Na/Fe/P/O/F is approximately the stoichiometric composition of Na_2_FePO_4_F, this is indicative of high purity of the product. The doped nitrogen content is estimated to be 1.6 wt%. The high‐resolution Fe 2p spectrum with the Fe 2p_3/2_ and Fe 2p_1/2_ doublets located at 711.2 and 724.6 eV, respectively (Figure 2d), is characteristic of Fe^2+^,^28^, ^34^ agreeing well with the bivalence state of iron in Na_2_FePO_4_F. Figure 2e displays the N 1s spectrum, three deconvoluted peaks centered at 398.3, 399.7, and 400.8 eV correspond to the pyridinic N, pyrrolic N, and graphitic N, respectively,^37^, ^38^ their content percentages are in sequence, 31.45, 35.23, and 33.32%, based on the area percentages of the corresponding fitted peaks. The formation of the C=N (pyridinic) and C—N (pyrrolic) bonds is further confirmed by the C 1s spectrum in Figure 2f. Noticeably, nitrogen doping can synchronously contribute electrons and create defects to the carbon lattice,^37^, ^39^ thus effectively facilitating the electronic/ionic transportation.

**Figure 3:**
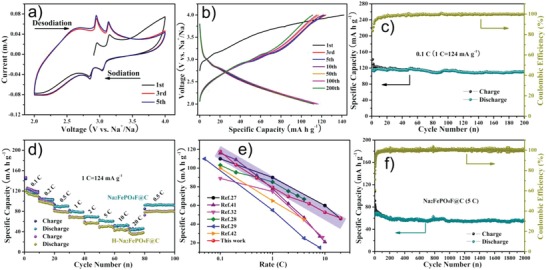
a) Cyclic voltammograms of the Na_2_FePO_4_F@C cathode at a scan rate of 0.1 mV s^−1^ in the voltage window of 2.0–4.0 V versus Na^+^/Na. b) Galvanostatic charge/discharge profiles and c) cycling performance of Na_2_FePO_4_F@C at a rate of 0.1 C between 2.0 and 4.0 V. d) Rate capability of Na_2_FePO_4_F@C in comparison with the counterpart containing higher Na_2_FePO_4_F content (H‐Na_2_FePO_4_F@C). e) Comparison of this work with the previously reported Na_2_FePO_4_F‐based SIB cathode materials. f) Long‐term cyclic stability of the Na_2_FePO_4_F@C electrode at 5 C.

For the evaluation of the electrochemical properties, the as‐prepared Na_2_FePO_4_F@C fiber membrane adherent on Al current collector was cut into suitable size and directly assembled into Na‐ion half cells as binder‐free working electrode. The mass loading density is in the range of 2.3–2.7 mg cm^−2^, which can be controlled by adjusting the electrospinning time. **Figure** **3**a shows the typical cyclic voltammetry (CV) curves of Na_2_FePO_4_F@C, two pairs of redox peaks situated at 3.04/3.13 and 2.85/2.94 V are ascribed to the two reversible phase transitions of Na_2_Fe^II^PO_4_F ↔ Na_1.5_FePO_4_F and Na_1.5_FePO_4_F ↔ NaFe^III^PO_4_F, respectively.^27^, ^28^ There are two crystallographic Na sites in the structure of Na_2_FePO_4_F, where one can be reversibly extracted due to its lower surrounding binding energy, while the other is electrochemically inert in order to maintain the layered framework (shown later).^33^, ^35^ It should be mentioned that the CV curves are almost overlapped excluding the first cycle, implying the good reversibility. The representative charge/discharge profiles at 0.1 C rate (1 C = 124 mA g^−1^) of the Na_2_FePO_4_F@C cathode are plotted in Figure 3b. In agreement with the above CV study, two voltage plateaus at 2.9 and 3.1 V are characterized and associated with the activation energies for Na^+^ migration along the [001] and [100] orientations (discussed later).^27^ The initial charge and discharge capacities are 142 and 116 mAh g^−1^, respectively, corresponding to a Coulombic efficiency (CE) of 81.7%, the capacity fading in the first cycle is mainly caused by the decomposition of electrolyte to form a solid‐electrolyte interface (SEI) film at the high voltage region. Subsequently, the voltage profiles maintain the similar shape and deliver a steady reversible capacity of ≈115 mAh g^−1^ up to 200 cycles. The achievable capacity is close to the theoretical value of 124 mAh g^−1^, validating the high utilization rate of active materials. The superb cyclic stability of Na_2_FePO_4_F@C is further demonstrated in Figure 3c, affording a discharge capacity of 111.1 mAh g^−1^ with a capacity retention of 96% after 200 cycles. Considering that the N‐doped carbon nanofiber matrix (without Na_2_FePO_4_F) contributes negligible capacity to the composite electrode in the potential range of 2.0–4.0 V versus Na^+^/Na (Figure S6, Supporting Information), the specific capacity values in this paper are calculated based on the active Na_2_FePO_4_F mass.

Actually the carbon content in the Na_2_FePO_4_F@C nanocomposite has been optimized to pursue the best Na‐storage performance. Figure S7 (Supporting Information) provides the SEM images of the sample with lower or higher Na_2_FePO_4_F loading (labeled as L‐Na_2_FePO_4_F@C or H‐Na_2_FePO_4_F@C), which also appear as a reticular morphology interlinked by nanofibers but many nanoparticles are exposed outside the fiber skeleton for H‐Na_2_FePO_4_F@C. TEM images (Figure S8, Supporting Information) further verify that the L‐Na_2_FePO_4_F@C contains insufficient Na_2_FePO_4_F nanograins whereas excess Na_2_FePO_4_F content in the H‐Na_2_FePO_4_F@C significantly drives the grains to grow bigger (some even to 30–50 nm). The Na_2_FePO_4_F contents in L‐Na_2_FePO_4_F@C, Na_2_FePO_4_F@C, and H‐Na_2_FePO_4_F@C are 69.3, 77.8, and 81.6 wt%, respectively, as determined by thermogravimetric analyses (TGA, Figure S9, Supporting Information) and element analyses (Table S1, Supporting Information). It is worth mentioning that the active mass loading in the Na_2_FePO_4_F@C cathode is comparable to those of the conventionally used SIB cathodes, benefiting from the binder‐free character of the Na_2_FePO_4_F@C electrode without any polymer binder or conductive carbon. Figure S10 (Supporting Information) gives the Raman spectra of the aforementioned three Na_2_FePO_4_F@C samples, the two main peaks located at ≈1340 and ≈1605 cm^−1^ are attributed to the disorder‐induced (D) band and graphitic‐induced (G) band of carbon, respectively.^37^, ^39^ The Gaussian fitting results indicate the amorphous nature of the carbon nanofibers since the intensity ratios of D mode to G mode (*I*_D_/*I*_G_) are in the range of 1.11–1.17, thereby the structural defects and vacancies can offer abundant diffusion channels for Na^+^ ions. In addition, the integrated area ratios of sp^3^ carbon to sp^2^ carbon (*A*_sp3_/*A*_sp2_) ranging from 32 to 40% manifest the high proportion of sp^2^ species and thus the good electronic conductivity.^34^, ^40^ This is solidly evidenced by the electrochemical impedance spectra (EIS) in Figure S11 (Supporting Information), revealing that the charge‐transfer resistance (*R*_ct_, evaluated from the depressed semicircle) of Na_2_FePO_4_F@C is much smaller than that of H‐Na_2_FePO_4_F@C (107 Ω mg^−1^ vs 169 Ω mg^−1^), with the apparent Na‐ion diffusion coefficient (*D*_Na_, calculation details are provided in the Supporting Information) being much higher (2.07 × 10^−13^ cm^2^ s^−1^ vs 1.67 × 10^−14^ cm^2^ s^−1^). As can be concluded, the homogeneous encapsulation of ultrasmall Na_2_FePO_4_F nanoparticles into carbon nanofibers could simultaneously boost the electrons transfer and sodium ions diffusion. Figure 3d depicts the rate capability of the Na_2_FePO_4_F@C cathode, with the testing rate progressively increasing from 0.1, 0.2, 0.5, 1, 2, 5, 10 to 20 C, corresponding reversible capacities of 117.8, 103.5, 89.6, 79.4, 69.7, 62.0, 52.7, and 46.4 mAh g^−1^ are successively gained. Impressively, when the rate is turned back to 0.5 C, the discharge capacity swiftly recovers to 92.2 mAh g^−1^, attesting the excellent tolerance ability for rapid Na‐insertion/extraction. By contrast, the H‐Na_2_FePO_4_F@C electrode delivers inferior rate performance due to its relatively sluggish reaction kinetics and poor structural stability caused by the larger Na_2_FePO_4_F nanoparticles that are not entirely enwrapped by carbon. Postmortem examinations (Figure S12, Supporting Information) were conducted to prove this viewpoint, the Na_2_FePO_4_F@C still sustains its original appearance and structure with nanodots uniformly embedded in nanofibers after the high‐rate cycling, while the H‐Na_2_FePO_4_F@C suffers from particles pulverization and aggregation issues after 100 cycles. The L‐Na_2_FePO_4_F@C electrode exhibits competitive rate performance to that of the Na_2_FePO_4_F@C (Figure S13, Supporting Information), but its actual energy density should be lower due to the limited active mass loading. Compared with the previously reported Na_2_FePO_4_F‐based Na‐storage cathode materials,^27^, ^28^, ^29^, ^32^, ^41^, ^42^ the superior rate capability of Na_2_FePO_4_F@C nanofibers is further highlighted in Figure 3e (especially considering the relatively high electrode mass loading of ≈2.5 mg cm^−2^ in this work than the commonly used 1–2 mg cm^−2^). Remarkably, Figure 3f displays the long‐term cycling performance of the Na_2_FePO_4_F@C cathode at a constant rate of 5 C, demonstrating an unprecedented capacity retention of 85% even after 2000 cycles. Moreover, the CE values approach 100% during the prolonged cycling, which is beneficial for the ultralong cycle life. The tailored ultrasmall Na_2_FePO_4_F nanoparticles exposing abundant active sites favor the easy access to Na^+^ ions and the sufficient utilization for Na‐storage, the construction of a robust 3D network by nanofibers benefits the electronic/ionic mobility and the architecture steadiness, and the confinement of active materials into porous carbon matrix effectively alleviates the side reactions with electrolyte,^36^, ^37^, ^39^ thus rendering the outstanding electrochemical performance in terms of high reversible capacity, exceptional rate capability, and distinguished cyclic stability.

**Figure 4:**
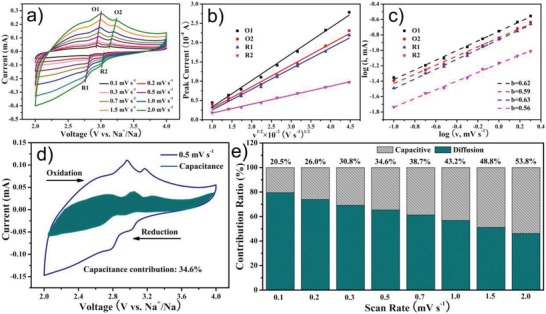
a) CV profiles at different scan rates ranging from 0.1 to 2.0 mV s^−1^, b) fitting curves between the peak currents (*i*_p_) and the square root of scan rates (*v* ^1/2^), and c) log*i*_p_ versus log*v* linear relationships of the Na_2_FePO_4_F@C cathode. d) CV profile at 0.5 mV s^−1^ illustrating the pseudocapacitance contribution (cyan region) to the total current, along with e) percentages of the capacitive response and the diffusion‐controlled behaviour in the Na_2_FePO_4_F@C electrode process at various scan rates.

The electrode process kinetics of Na_2_FePO_4_F@C is then investigated to explain its fascinating Na‐storage performance. **Figure** **4**a presents the CV profiles of Na_2_FePO_4_F@C recorded at various sweep rates from 0.1 to 2.0 mV s^−1^, as the scan rate increases, the redox peak currents increase prominently, while the potential intervals between the oxidation (O1 and O2) and reduction (R1 and R2) peaks increase slightly, this implies the minor polarization of the Na_2_FePO_4_F@C cathode. The Na‐ion diffusivity can be determined according to the Randles–Sevcik equation of *i*_p_ = 2.69 × 10^5^ *n* ^3/2^ *AD*_Na_^1/2^ *C*_Na_ *v* ^1/2^, where *i*_p_ (A) is the peak current, *n* is the electron transfer number per molecule (*n* = 1 in this system), *A* (cm^2^) is the contacting area between electrode and electrolyte, *C*_Na_ is the Na ions concentration in the electrode (≈3.8 × 10^−3^ mol cm^−3^), and *v* (V s^−1^) is the scan rate.^34^, ^43^ Figure 4b plots the fitting curves between the peak currents and the square root of scan rates, as calculated, the *D*_Na_ values for the O1, O2, R1, and R2 peaks are 7.18 × 10^−11^, 4.69 × 10^−11^, 4.36 × 10^−11^, and 8.21 × 10^−12^ cm^2^ s^−1^, respectively. The smaller *D*_Na_ of the O2/R2 redox couple suggests that the reaction at high potential needs more activation energy. Notably, the achieved Na^+^ diffusion coefficient of Na_2_FePO_4_F@C compares favorably with those of other reported Na_2_FePO_4_F‐based cathodes in Na‐ion batteries (mostly at magnitude of ≈10^−12^–10^−15^ cm^2^ s^−1^).^27^, ^28^, ^41^ This is responsible for the high rate performance. Additionally, considering that the *D*_Na_ obtained based on CV data is a little larger than that determined from the EIS result, the pseudocapacitive behavior in the electrode should be further identified. The capacitance contribution of battery chemistry can be estimated according to the relation of *i*_p_ = *av^b^* or log*i*_p_ = *b* × log*v* + log*a*, of which *a* and *b* are variable parameters. A *b* value of 0.5 indicates a diffusion‐dominated process, whereas a *b* value of 1.0 signifies a surface‐controlled capacitive process.^44^, ^45^ As calculated from the slopes of log*i*_p_ versus log*v* linear relationships (Figure 4c), the *b* values for the O1, O2, R1, and R2 peaks are 0.62, 0.59, 0.63, and 0.56, respectively. This means that a combination of the diffusion‐limited and capacitive behaviors synergistically contributes to the charge storage process, enabling the fast reaction kinetics. Furthermore, the capacitance response in the Na_2_FePO_4_F@C electrode process can be quantified by dividing the current (*i*) at a fixed potential (*V*) into capacitive effect (*k*_1_ *v*) and diffusion‐controlled intercalation (*k*_2_ *v* ^1/2^) using the formula of *i*(V) = *k*_1_ *v* + *k*_2_ *v* ^1/2^.^45^, ^46^ Figure 4d shows the representative CV profile scanned at 0.5 mV s^−1^ with around 34.6% of the total current deriving from the pseudocapacitance contribution (cyan area). As expected, the capacitive effect gradually strengthens when increasing the sweep rate (Figure 4e), and the desirable capacitive behavior is originated from the favorable heterostructure incorporating nanoscale particles, carbon coating, and porosity.

**Figure 5:**
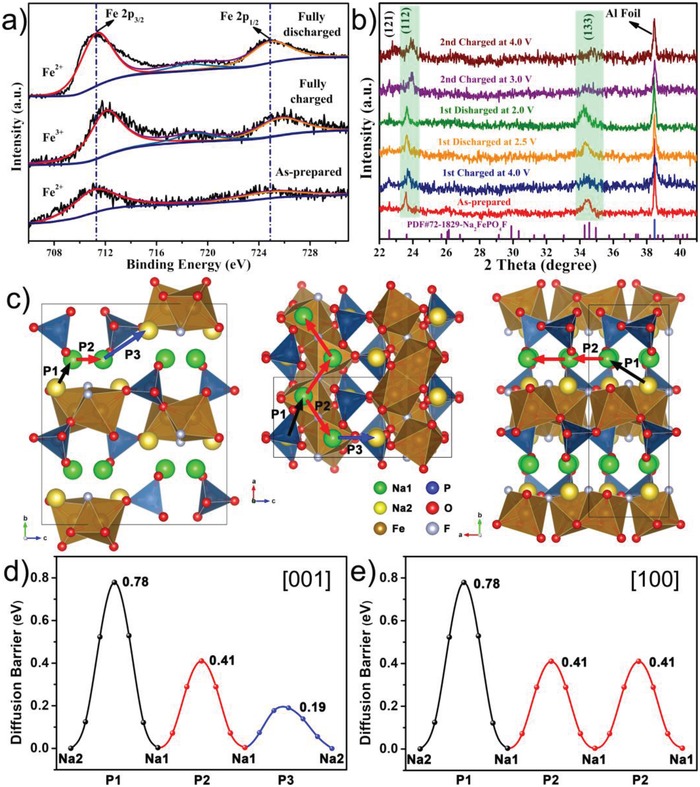
a) Ex situ Fe 2p XPS spectra of the Na_2_FePO_4_F@C cathode at the pristine, fully charged (4.0 V), and fully discharged (2.0 V) states in the first cycle. b) Ex situ XRD patterns of the Na_2_FePO_4_F@C electrode at selected charged/discharged stages in the initial two cycles. c) Schematic crystal structure of Na_2_FePO_4_F illustrating the possible Na^+^ migration pathways following Na2→Na1 (P1), Na1→Na1 (P2), and Na1→Na2 (P3) along the [001] (*c*‐axis) and [100] (*a*‐axis) directions, as well as the d,e) corresponding migration energy barriers.

To better understand the Na‐storage mechanism of the Na_2_FePO_4_F@C cathode, ex situ XPS and XRD were carried out. As shown in **Figure** **5**a, the fresh electrode displays characteristic Fe 2p signals of Fe^2+^; upon fully charged (4.0 V), the Fe 2p doublets move to higher binding energy along with the appearance of a satellite peak positioned between Fe 2p_3/2_ and Fe 2p_1/2_, indicating the oxidation state of Fe^3+^;^28^, ^47^, ^48^ when fully discharged (2.0 V), the Fe 2p peaks move back to the original positions of Fe^2+^. The above observations are consistent with the transition of Fe^2+^/Fe^3+^ redox couple during the reversible phase transformation of Na_2_FePO_4_F ↔ NaFePO_4_F. Figure 5b presents the structural evolution of the electrode material during repeated cycling, it is concluded that the diffraction peaks of (112) and (133) shift toward higher 2θ angles upon charging and recover to the pristine angles upon discharging in a quite symmetric way, demonstrating the highly reversible Na‐extraction/insertion reaction accompanied by the contraction/expansion of crystal cell. The Le Bail refined XRD results of the fully charged/discharged electrodes are given in Figure S14 (Supporting Information), which further attest the great reversiblity of crystal structure and the small cell volume change of 4.2% (by comparing their cell parameters with those of the original material, Table S2, Supporting Information). In addition, the intensities of the diffraction peaks generally weaken when charging and strengthen when discharging, reflecting the reversible phase transitions. To deeply probe the Na^+^ migration mechanism in the Na_2_FePO_4_F crystal, first‐principles computations based on the density functional theory (DFT) were performed. As elucidated in Figure 5c, three possible Na‐ion migration paths following Na2→Na1 (P1), Na1→Na1 (P2), and Na1→Na2 (P3) are rationally built, note that the crystallographic Na1 site should be occupied in order to maintain the stability of layered structure, whereas the Na2 site could be empty for ion extraction due to its smaller bond populations. The corresponding Na^+^ migration energy barrier profiles calculated based on the climbing‐image nudged elastic band (cNEB) method are plotted in Figure 5d,e. Along the [001] direction (*c*‐axis), the diffusion barriers for the routes of P1, P2, and P3 are 0.78, 0.41, and 0.19 eV, respectively; along the [100] direction (*a*‐axis), only the routes of P1 and P2 are exhibited; Na^+^ could hardly migrate along the [010] direction (*b*‐axis) which is perpendicular to the PO_4_‐FeO_4_F_2_ layers. The energy barriers for Na^+^ ion diffusion in the Na_2_FePO_4_F lattice are much lower than those of most SIB cathode materials,^49^, ^50^ accounting for the decent reaction dynamics and the outstanding rate performance.

**Figure 6:**
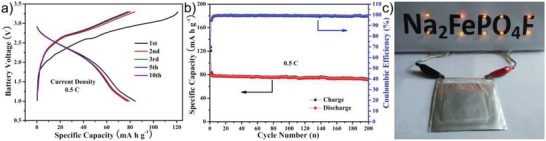
a) Galvanostatic charge/discharge curves and b) cycling performance of the pouch‐type sodium‐ion full battery employing the Na_2_FePO_4_F@C nanofibers cathode and the carbon nanofibers anode examined at 0.5 C rate in the voltage scope of 1.0–3.3 V (based on the cathode active mass). c) Digital photo of the soft package full battery powering ten LEDs.

The success of Na_2_FePO_4_F@C nanofibers in Na‐ion half cells strongly encourages us to further explore their practicability in sodium‐ion full batteries. The pure carbon nanofibers adherent on Al foil were adopted as a binder‐free anode to match with the Na_2_FePO_4_F@C binder‐free cathode. The Na‐storage behavior of the carbon anode in the potential range of 0.01–2.0 V versus Na^+^/Na is provided in Figure S15 (Supporting Information), showing a stable reversible capacity of ≈205 mA h g^−1^ for 200 cycles at 100 mA g^−1^. Therefore, the active mass ratio of anode to cathode was carefully balanced to be 1:1.8. **Figure** **6**a,b depicts the electrochemical performance of the assembled pouch‐type full battery evaluated in the voltage window of 1.0–3.3 V, delivering an initial discharge capacity of 84.5 mAh g^−1^ at 0.5 C with the operating voltage of about 2.5 V (based on the active mass on the cathode side), as well as an admirable capacity retention of 84.5% over 200 cycles with the CE values of around 99%. In this case, the energy density of the full battery can reach as high as 135.8 Wh kg^−1^ based on the total mass of cathode and anode active materials. More impressively, the Na_2_FePO_4_F@C//hard carbon pouch battery can continuously light up ten light‐emitting diodes (LEDs, Figure 6c), corroborating the great potentiality for practical applications.

## 3 Conclusion

In summary, a high‐performance SIB cathode material was realized by homogeneously encapsulating the smallest Na_2_FePO_4_F nanoparticles (≈3.8 nm) ever reported into porous N‐doped carbon nanofibers. The as‐prepared Na_2_FePO_4_F@C fiber membrane tightly adhered to aluminum foil featured great flexibility and served directly as a binder‐free cathode for Na‐ion batteries, showing the advantages of enhanced electrochemical activity, accelerated electrons/ions transportation, and reinforced structural stability. Consequently, intriguing Na‐storage performance of high reversible capacity (117.8 mAh g^−1^ at 0.1 C), superior rate capability (46.4 mAh g^−1^ at 20 C), and unprecedentedly long cycling life (85% capacity retention over 2000 cycles) was exhibited. In addition, the reaction kinetics and mechanism of the Na_2_FePO_4_F@C cathode were systematically investigated by techniques of CV study, ex situ XPS, XRD, and first‐principles computations, demonstrating the highly reversible phase transition of Na_2_Fe^II^PO_4_F ↔ NaFe^III^PO_4_F with facilitated Na^+^ diffusion dynamics and low ion‐migration energy barriers, the pseudocapacitive behavior is also identified to benefit the high‐rate performance. More significantly, pouch‐type sodium‐ion full batteries were constructed by coupling the Na_2_FePO_4_F@C nanofibers cathode with the carbon nanofibers anode, delivering a promising energy density of 135.8 Wh kg^−1^ along with a high durability of 84.5% capacity retention after 200 cycles. To our knowledge, such distinguished overall performance has scarcely been achieved for the Na_2_FePO_4_F‐based Na‐storage cathode materials and is also prominent among all the reported SIB cathodes to date. The controllable/green fabricating approach, the fascinating electrochemical performance, and the deep understanding of electrode process illuminate bright prospects for the Na_2_FePO_4_F@C nanofibers as practicable SIB cathode materials.

## 4 Experimental Section

*Materials Synthesis*: The Na_2_FePO_4_F@C nanofibers were fabricated via a simple electrospinning method followed by a two‐step heat treatment. In a typical preparation, 2.1 g PVP (average *M*_w_ = 1 300 000, Aladdin) was first dissolved in 15 mL deionized water under magnetic stirring at room temperature. Then, stoichiometric quantities (4 mmol) of citric acid (99.5%, Aladdin), Fe(CH_3_COO)_2_ (97%, J&K), NaH_2_PO_4_ (99.5%, Aladdin), and NaF (99.99%, Macklin) were added to the above solution in order, and vigorously stirred at room temperature for another 2 h to form an uniform precursor spinning solution.

The obtained solution was transferred into a 10 mL plastic syringe furnished with a 21‐gauge needle. An aluminum foil as the collector was used to gather the fibers. The electrostatic voltage applied between the collector and the needle was 17 kV, and the distance was set to be 15 cm. The flowing rate of fluid was 10 µL min^−1^ controlled by a syringe pump. After continuous electrospinning at 40 °C for 36 h, fiber membrane with a thickness of about 100–120 µm can be achieved.

The as‐spun fiber film tightly adhered to aluminum foil was first stabilized at 300 °C for 1 h and then thermally treated at 600 °C for 6 h under N_2_ environment to get the target NaFe_2_PO_4_F@C nanofibers. The temperature ramping rate was 5 °C min^−1^. For comparison, counterpart samples with lower or higher Na_2_FePO_4_F content (designated as L‐Na_2_FePO_4_F@C or H‐Na_2_FePO_4_F@C), and N‐doped carbon nanofibers without Na_2_FePO_4_F by adjusting the dosage of citric acid/Fe(CH_3_COO)_2_/NaH_2_PO_4_/NaF to 3, 5, or 0 mmol, respectively (the other conditions were kept constant) were also prepared. In addition, the annealing temperature (500 or 700 °C) and time (3 or 9 h) to optimize the synthesis conditions were modulated.

*Materials Characterizations*: The crystal structure and phase purity of the as‐prepared samples were analyzed by XRD (Rigaku D/Max‐2500) using Cu Kα radiation (λ = 1.5418 Å) in the 2θ range of 10°–70°. The morphology and microstructure were investigated by SEM (JEOL JSM7500F) and TEM (TECNAI G2 20). Thermogravimetric analysis‐differential scanning calorimetry (TG‐DSC) analyzer (STA 449 F3) was used to determine the carbon content of composites (heating from room temperature to 650 °C in air). Raman spectra were obtained on a confocal Raman spectrometer (ThermoFisher Scientific) using argon‐ion laser excitation at 532 nm. XPS was performed on a PHI 1600 ESCA spectrometer (Perkin‐Elmer). N_2_ adsorption–desorption measurement was carried out with an ASAP 2460 instrument (MicroActive) at 77 K.

*Electrochemical Measurements*: The electrochemical properties were evaluated with CR2032 coin cells assembled in a glove box (Mikrouna Universal 2440/750) filled with ultrapure argon. The as‐synthesized Na_2_FePO_4_F@C film attached on Al foil was cut into a suitable shape and directly applied as working electrode without using conductive additives or binder. The electrode was dried overnight at 80 °C in vacuum and the mass loading density was ≈2.5 mg cm^−2^. Na metal foil was used as the counter/reference electrode and glass microfiber was adopted as the separator. The electrolyte was 1 m NaClO_4_ in propylene carbonate (PC) solution with addition of 5 vol% fluoroethylene carbonate (FEC). Galvanostatic charge/discharge tests were conducted on a LAND battery tester (CT2001A) in the potential scope of 2.0–4.0 V (vs Na^+^/Na) at different rates. CV profiles at diverse scanning speeds (0.1–2.0 mV s^−1^) between 2.0 and 4.0 V were recorded on a CHI660C electrochemical workstation (Chenhua, Shanghai), and EIS were collected at open‐circuit voltage (OCV) in the frequency range of 100 kHz to 100 mHz with the AC disturbance signal of 5.0 mV (the electrodes for EIS measurements were mass normalized). In order to inspect the cycled active materials by XRD, SEM, TEM, and XPS, the electrodes were taken out from cells in a glove box and then repeatedly rinsed with dimethyl carbonate (DMC) solution to remove the residual electrolyte.

For sodium‐ion full batteries, the Na_2_FePO_4_F@C nanofibers cathode was paired with the pure carbon nanofibers anode. The electrochemical performance was examined within the voltage window of 1.0–3.3 V. To balance the capacity, the active mass ratio of anode to cathode was set to be 1:1.8. Furthermore, the specific capacity and current density of full batteries were normalized to the mass of cathode active materials.

*Computational Details*: Ab initio simulations based on DFT were performed by Vienna ab initio simulation package (VASP) using projector augmented wave (PAW) method with Perdew–Burke–Ernzerhof (PBE) function. The energy cutoff was set to 500 eV and the (4 × 1 × 2) k‐point meshes were used for Na_2_FePO_4_F. The convergence tolerance for the force and energy during relaxation were 0.01 eV Å^−1^ and 10^−5^ eV, respectively. The cNEB method was adopted to simulate the Na^+^ migration paths and barriers.

## Conflict of Interest

The authors declare no conflict of interest.

## Supporting information

Supplementary
