# Supplementary material for: Realizing a High‐Performance Na‐Storage Cathode by Tailoring Ultrasmall Na2FePO4F Nanoparticles with Facilitated Reaction Kinetics
Source: Adv Sci (Weinh). 2019 May 7;6(13):1900649. doi: 10.1002/advs.201900649 (PMC6662290; doi:10.1002/advs.201900649)
Supplement: Supplementary file 1 — Supplementary [file ADVS-6-1900649-s001.pdf]

Copyright WILEY-VCH Verlag GmbH & Co. KGaA, 69469 Weinheim, Germany, 2019.

Supporting Information

for *Adv. Sci.,* DOI: 10.1002/advs.201900649

Realizing a High-Performance Na-Storage Cathode by Tailoring Ultrasmall Na_2FePO_4F Nanoparticles with Facilitated Reaction Kinetics

Fanfan Wang, Ning Zhang, Xudong Zhao, Lixuan Wang, Jian Zhang, Tianshi Wang, Fanfan Liu, Yongchang Liu, and Li-Zhen Fan**

Copyright WILEY-VCH Verlag GmbH & Co. KGaA, 69469 Weinheim, Germany, 2019.

Supporting Information

Realizing a High-Performance Na-Storage Cathode by Tailoring Ultrasmall Na_2FePO_4F Nanoparticles with Facilitated Reaction Kinetics

Fanfan Wang,+ Ning Zhang,+ Xudong Zhao, Lixuan Wang, Jian Zhang, Tianshi Wang, Fanfan Liu, Yongchang Liu, and Li-Zhen Fan**

F. Wang, Dr. X. Zhao, Dr. J. Zhang, Dr. T. Wang, Dr. F. Liu, Prof. Y. C. Liu, Prof. L.-Z. Fan
Beijing Advanced Innovation Center for Materials Genome Engineering
Institute for Advanced Materials and Technology
University of Science and Technology Beijing, Beijing 100083, China
E-mail: liuyc@ustb.edu.cn; fanlizhen@ustb.edu.cn

Prof. N. Zhang
College of Chemistry & Environmental Science, Hebei University, Baoding 071002, China

Prof. N. Zhang, Prof. Y. C. Liu
Key Laboratory of Advanced Energy Materials Chemistry (Ministry of Education), Nankai University, Tianjin 300071, China

L. Wang
School of Electrical Engineering and Automation, Tianjin Polytechnic University, Tianjin 300387, China

+F. Wang and N. Zhang contributed equally to this work

Supplementary Figures and Tables

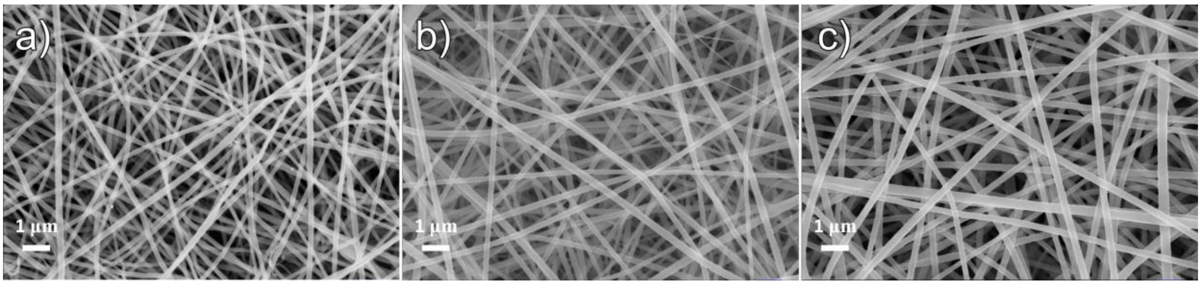

Figure S1. SEM images of the electrospun nanofibers using different dosages of raw materials: a) 3 mmol, b) 4 mmol, c) 5 mmol of $Fe(CH_3COO)_2$, NaH_2PO_4, and NaF.

The as-spun nanofibers are smooth and continuous, and are interlinked into a three-dimensional network. As the molar amount of the raw materials increases from 3 to 5 mmol, the diameters of the obtained nanofibers gradually increase.

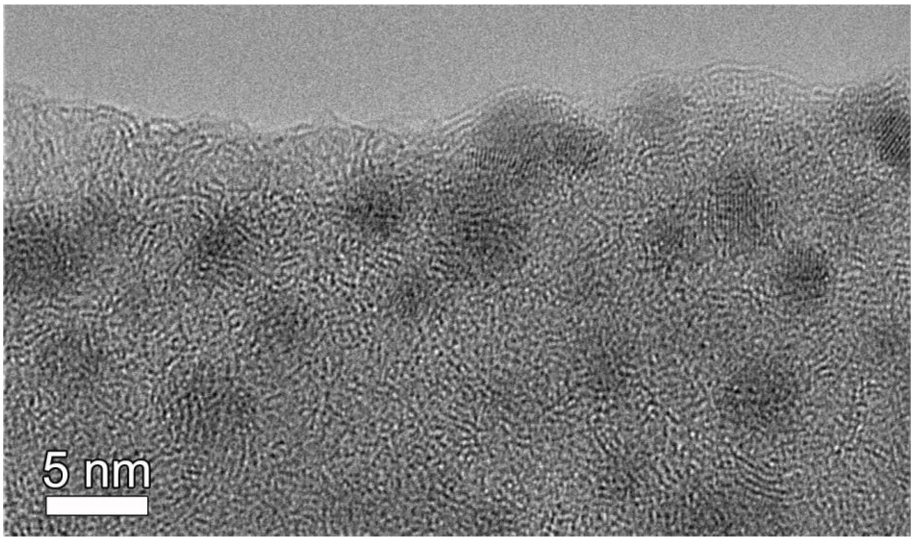

Figure S2. High magnification TEM image of the Na_2FePO_4F@C nanofibers.

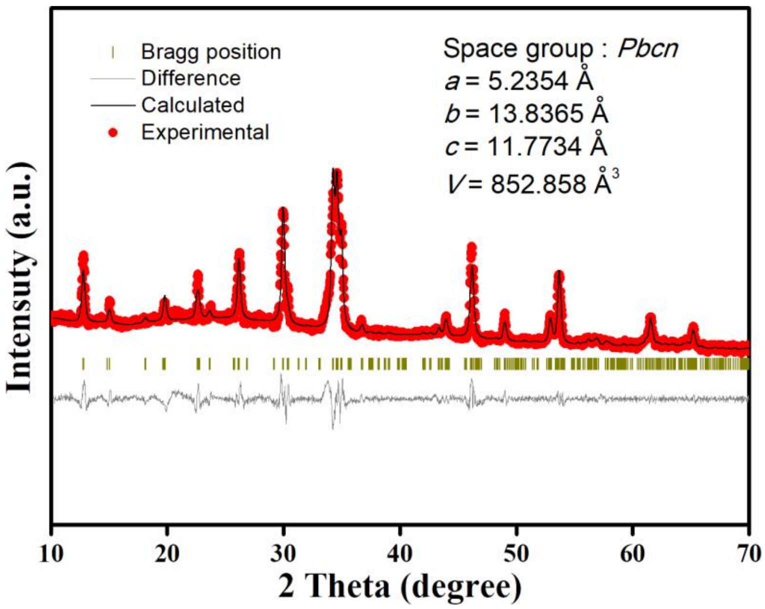

Figure S3. XRD pattern and Le Bail refinement result of the Na_2FePO_4F@C sample annealed at 600 °C for 6 h.

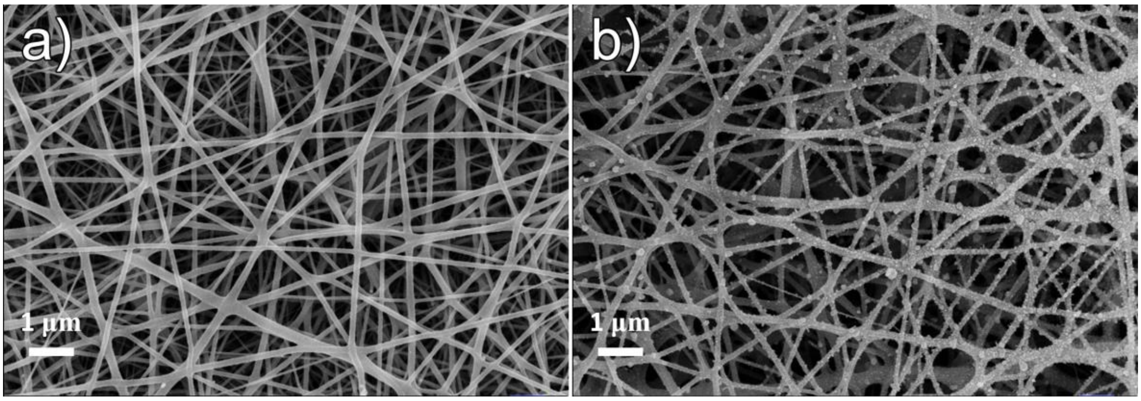

Figure S4. SEM images of the contrast samples with different calcination temperatures of a) 500 °C and b) 700 °C. The other synthetic conditions are the same as those of the Na_2FePO_4F@C nanofibers.

A low annealing temperature of 500 °C leads to a reticular morphology interlinked by smooth nanofibers; while a high temperature of 700 °C results in rough nanofibers caused by the growth and aggregation of Na_2FePO_4F particles (high calcination temperature increases the surface energy of Na_2FePO_4F nuclei).

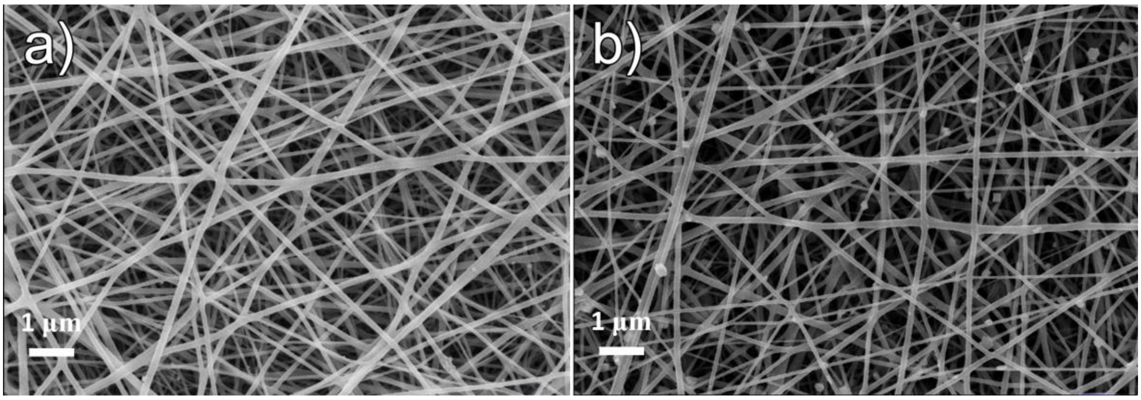

Figure S5. SEM images of the contrast samples with different calcination times of a) 3 h and b) 9 h. The other synthetic conditions are the same as those of the Na_2FePO_4F@C nanofibers.

A short heating time of 3 h leads to a reticular morphology interlinked by smooth nanofibers; while a long sintering time of 9 h results in rough nanofibers caused by the growth and aggregation of Na_2FePO_4F particles (long calcination time increases the surface energy of Na_2FePO_4F nuclei).

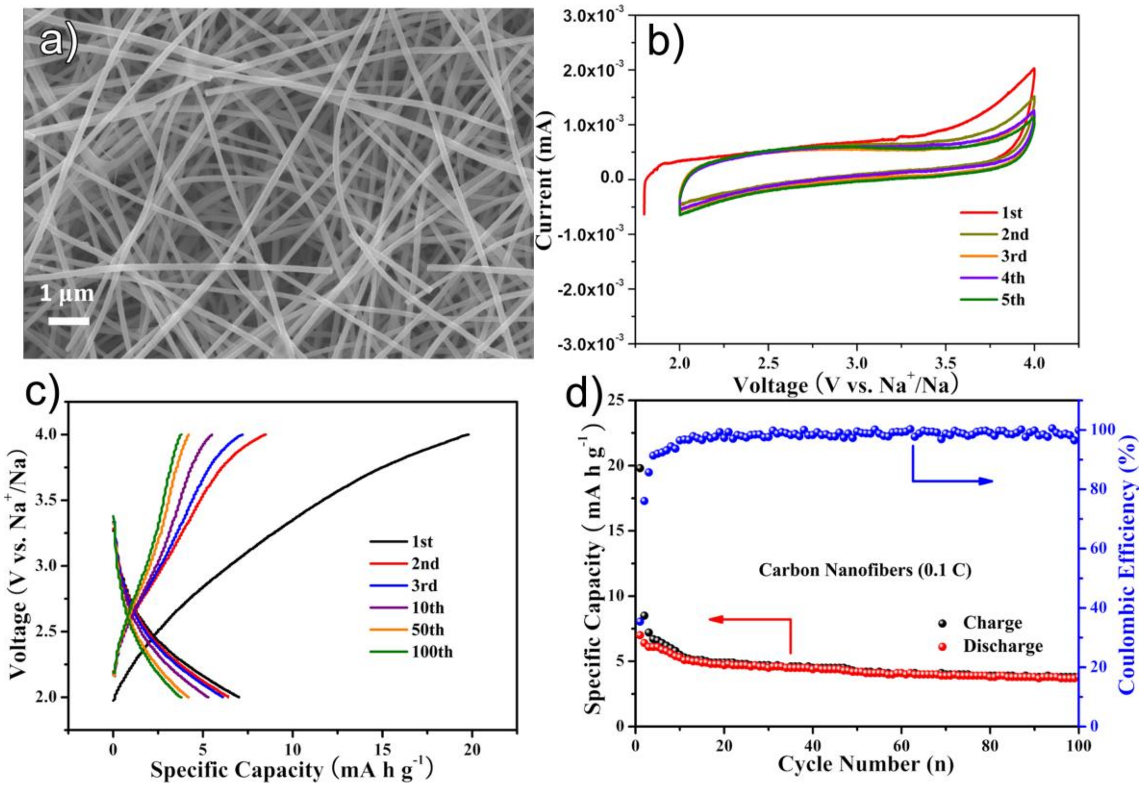

Figure S6. a) SEM image, b) CV curves at a scan rate of 0.1 mV s^{-1}, c) galvanostatic charge/discharge profiles and d) cycling performance at 0.1 C (1 C = 124 mA g^{-1}) in the potential window of 2.0-4.0 V vs. Na^{+}/Na of the N-doped carbon nanofibers (without Na_2FePO_4F). Note: the mass loading density of the carbon electrode is ∼0.6 mg cm^{-2}, about 24% of that for the Na_2FePO_4F@C electrode, following the carbon content in the Na_2FePO_4F@C composite (shown later in Figure S9).

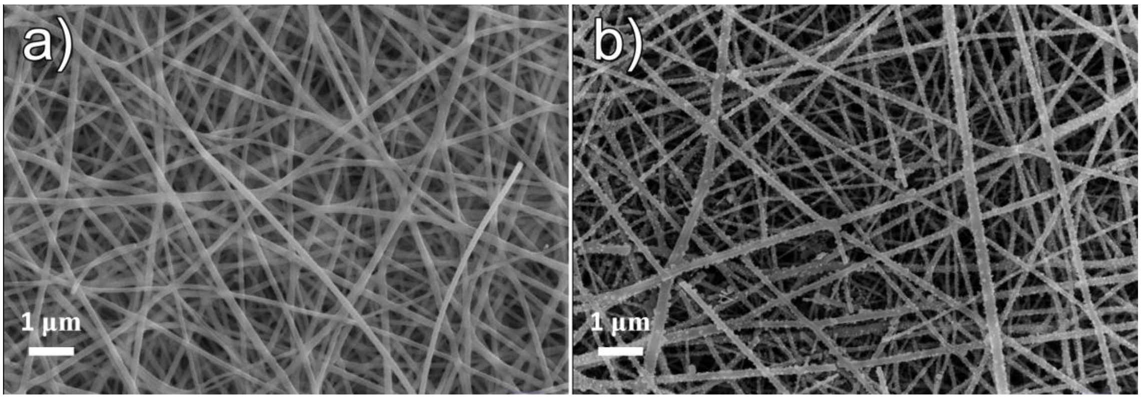

Figure S7. SEM images of the counterpart samples with a) lower Na_2FePO_4F content (L-Na_2FePO_4F@C) and b) higher Na_2FePO_4F content (H-Na_2FePO_4F@C).

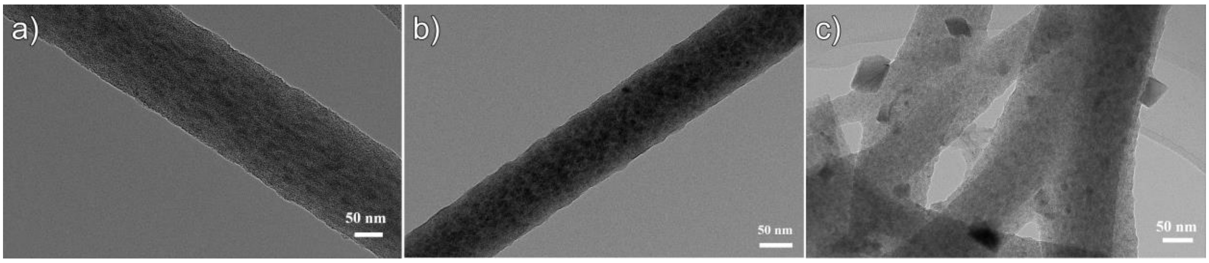

Figure S8. TEM images of the a) L-Na_2FePO_4F@C, b) Na_2FePO_4F@C, and c) H-Na_2FePO_4F@C nanofibers.

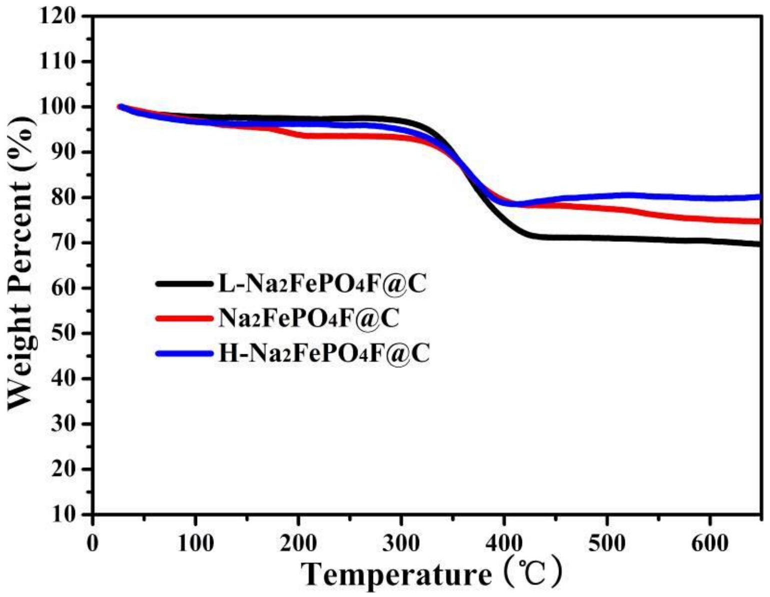

Figure S9. TGA curves of the L-Na_2FePO_4F@C, Na_2FePO_4F@C, and H-Na_2FePO_4F@C samples tested in ambient air from room temperature to 650 °C with a heating rate of 5 °C min^{-1}.

The tiny weight loss observed until 300 °C is caused by the evaporation of adsorbed water. This means that the composites are stable in air up to 300 °C. The fast weight loss from 300 to 430 °C is attributed to the carbon combustion ($C + O_2 \rightarrow CO_2\uparrow$). The subsequent slight weight increase is assigned to the oxidation of Fe^{2+} to Fe^{3+} ($12Na_2FePO_4F + 3O_2 \rightarrow 12NaF + 4Na_3Fe_2(PO_4)_3 + 2Fe_2O_3$).[1] The Na_2FePO_4F contents in L-Na_2FePO_4F@C, Na_2FePO_4F@C, and H-Na_2FePO_4F@C are determined to be approximately 69.3 wt%, 77.8 wt%, and 81.6 wt%, respectively.

Table S1. Element analyses of the as-prepared Na_2FePO_4F@C samples.

Sample	Element (wt%)		
	C	N	Na_2FePO_4F
L-Na_2FePO_4F@C	28.1	2.0	69.9
Na_2FePO_4F@C	20.3	1.5	78.2
H-Na_2FePO_4F@C	16.5	1.2	82.3

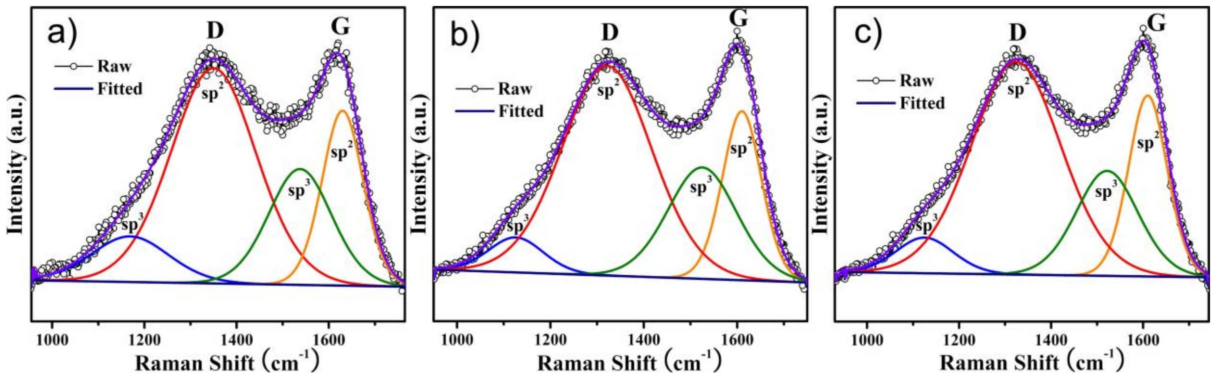

Figure S10. Raman spectra with deconvoluted peaks of the a) L-Na_2FePO_4F@C, b) Na_2FePO_4F@C, and c) H-Na_2FePO_4F@C nanofibers.

According to the Gaussian fitting,[2,3] the two broad Raman peaks of each sample can be deconvoluted into four peaks, of which the two peaks located at ~1335 and ~1610 cm^{-1} are ascribed to the sp^2-type D band and G band, respectively, the other two at ~1140 and ~1520 cm^{-1} correspond to the sp^3-type carbon. Therefore, the intensity ratios of D band to G band (I_D/I_G) are 1.17, 1.15, and 1.11 for L-$NaFePO_4$@C, $NaFePO_4$@C, and H-$NaFePO_4$@C, respectively. This implies the amorphous structure of the carbon nanofibers with abundant defects and vacancies,[4] offering more open channels for Na^+ diffusion.

The integrated area ratio of sp^3 carbon to sp^2 carbon (A_{sp3}/A_{sp2}) also provides useful information on the nature of carbon nanofibers. Based on the fitting results, the A_{sp3}/A_{sp2} values are 40.8%, 37.7%, and 32.1% for L-$NaFePO_4$@C, $NaFePO_4$@C, and H-$NaFePO_4$@C, respectively, indicating a high proportion of the sp^2-type carbon. This is beneficial for the high electronic conductivity.[3,5]

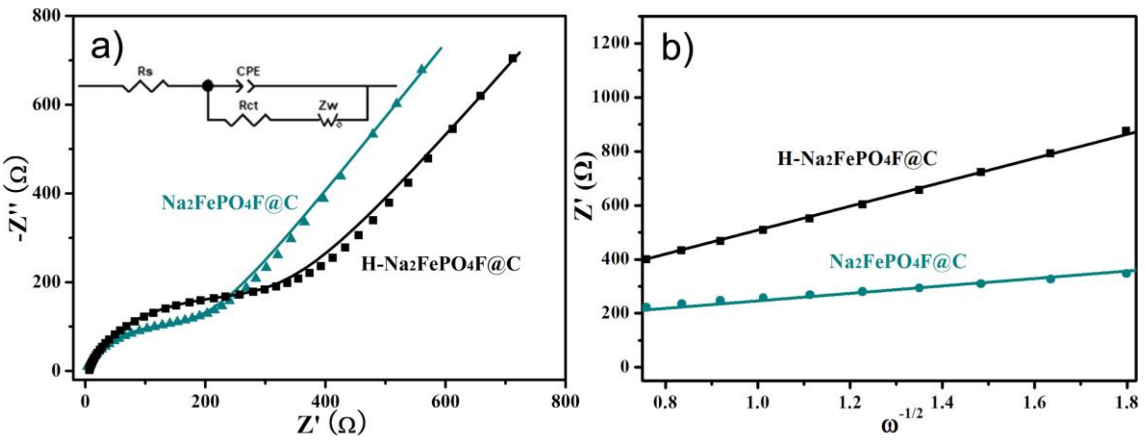

Figure S11. a) Nyquist dots of the Na_2FePO_4F@C and H-Na_2FePO_4F@C electrodes, along with the simulated curves based on the inset equivalent circuit. b) Linear relationships of the real parts of impedance (Z′) versus the reciprocal square root of angular frequency (ω) in the low frequency region.

The Nyquist plots in Figure S11a are composed of a depressed semicircle from high to medium frequency followed by a slope line in the low frequency region. Where the former is related to the charge-transfer resistance (R_{ct}) at the electrode/electrolyte interfaces, the latter refers to the Warburg impedance (Z_w) associated with Na^+ diffusion in the electrodes.[6,7] Based on the simulation results, the R_{ct} value of Na_2FePO_4F@C (210 Ω) is much smaller than that of H-Na_2FePO_4F@C (331 Ω). Considering the same loading density (2.5 mg cm^{-2}) and size (φ10 mm round slice) of the two electrodes, the mass normalized impedances are 107 Ω mg^{-1} for Na_2FePO_4F@C and 169 Ω mg^{-1} for H-Na_2FePO_4F@C, respectively.

In addition, the apparent diffusion coefficient of Na^+ (D_{Na}) can be estimated from the low frequency region according to the following equation (S1):

$$D_{Na} = \frac{R^2T^2}{2A^2n^4F^4C^2\sigma^2} \qquad \text{(S1)}$$

where R is the gas constant (8.314 J mol^{-1} K^{-1}), T is the absolute temperature (K), A is the contacting area of electrode with electrolyte (cm^2), n is the number of transferred electrons

during the redox reaction (n = 1 in this system), F is the Faraday constant (96500 C mol^{-1}), C is the concentration of sodium ions in the cathode (3.8 × 10^{-3} mol cm^{-3}), and σ is the Warburg factor. σ can be determined from the slope of Z′ vs. $\omega^{-1/2}$ lines (Figure S11b) according to the equation (S2):[1,7]

$$Z' = R_s + R_{ct} + \sigma\omega^{-1/2} \qquad \text{(S2)}$$

As calculated, the σ value of the Na_2FePO_4F@C electrode (138.4 Ω s$^{-1/2}$) is lower than that of the H-Na_2FePO_4F@C electrode (487.5 Ω s$^{-1/2}$), reflecting a higher D_{Na} value of the Na_2FePO_4F@C (i.e. 2.07 × 10^{-13} cm^2 s^{-1} vs. 1.67 × 10^{-14} cm^2 s^{-1}).

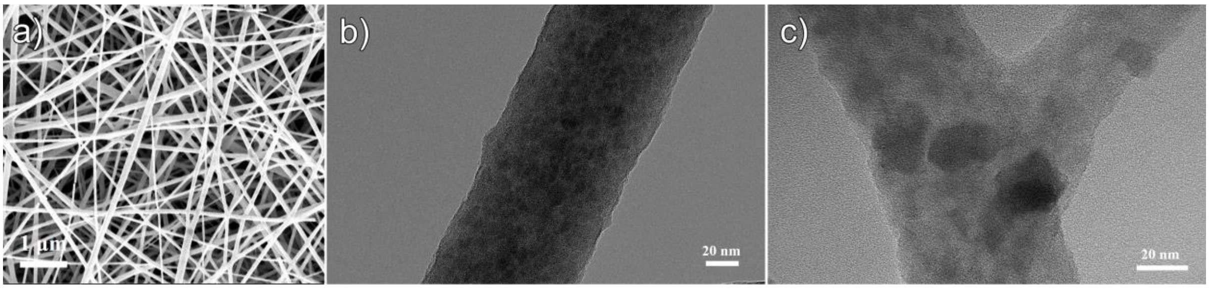

Figure S12. a) SEM and b) TEM images of the Na_2FePO_4F@C electrode material at the 2.0 V discharged state after 100 cycles. c) TEM image of the H-Na_2FePO_4F@C electrode material at the 2.0 V discharged state after 100 cycles. Testing program see Figure 3d.

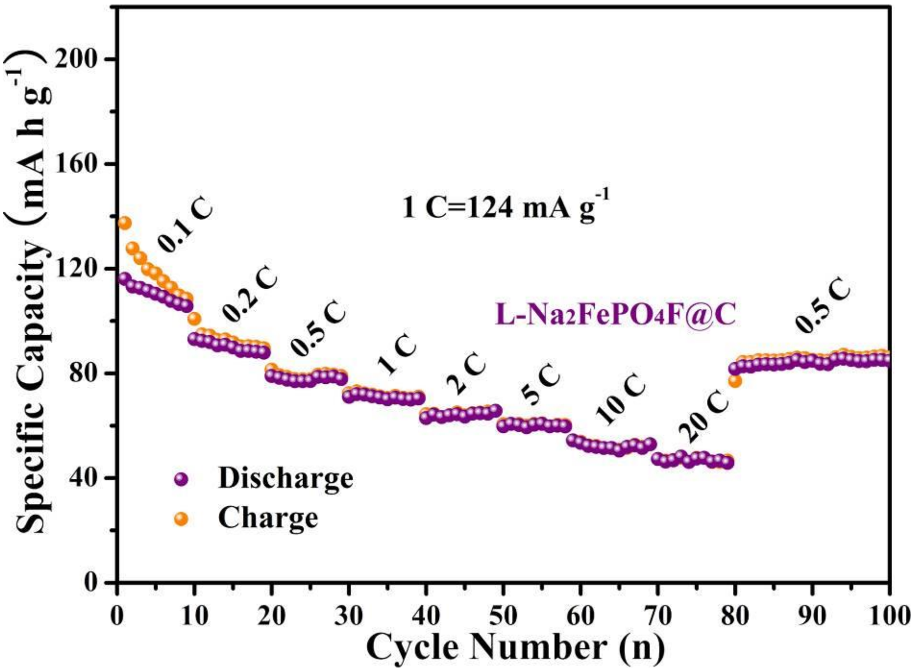

Figure S13. Rate capability of the L-Na_2FePO_4F@C electrode.

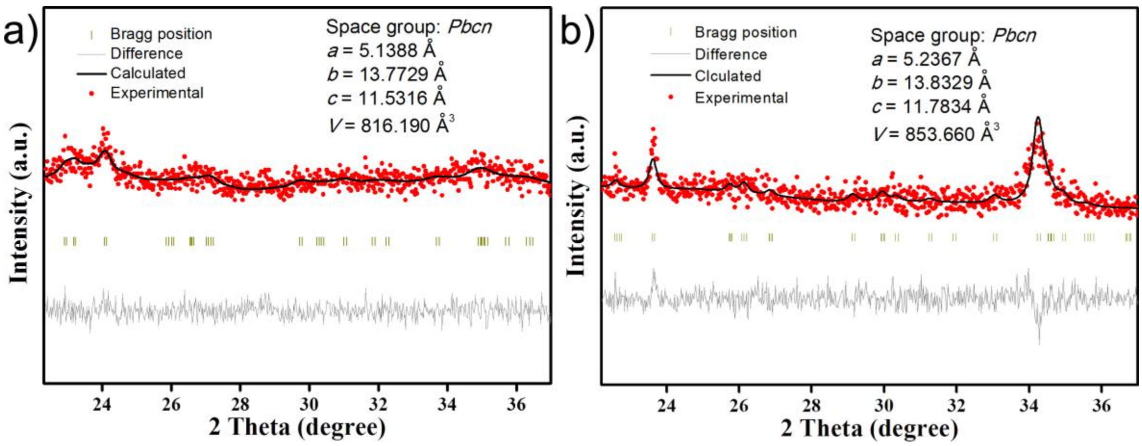

Figure S14. Le Bail refined XRD results of the fully a) charged and b) discharged electrodes.

Table S2. Lattice parameters and cell volume of the original sample and the charged/discharged products.

State	*a* (Å)	*b* (Å)	*c* (Å)	*V* (Å^3)	Δ*V* *
**Pristine**	5.2354	13.8365	11.7734	852.858	-
**Charged**	5.1388	13.7729	11.5316	816.190	4.2%
**Discharged**	5.2367	13.8329	11.7834	853.660	0.09%

*Note: ΔV is calculated based on the cell volume of pristine sample.

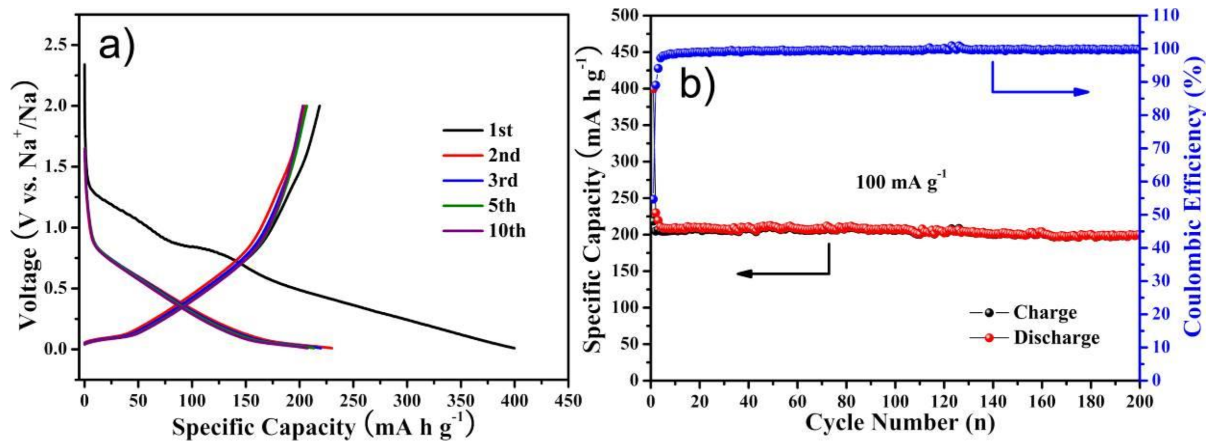

Figure S15. a) Galvanostatic charge/discharge profiles and b) cycling performance of the pure carbon nanofibers anode measured at a current density of 100 mA g^{-1} in the potential window of 0.01-2.0 V vs. Na^+/Na.

Supplementary References

[1] X. Deng, W. Shi, J. Sunarso, M. L. Liu, Z. P. Shao, *ACS Appl. Mater. Interfaces* **2017**, *9*, 16280-16287.

[2] A. Sadezky, H. Muckenhuber, H. Grothe, R. Niessner, U. Poschl, *Carbon* **2005**, *43*, 1731-1742.

[3] Y. C. Liu, N. Zhang, F. Wang, X. Liu, L. F. Jiao, L.-Z. Fan, *Adv. Funct. Mater.* **2018**, *28*, 1801917.

[4] J. Ding, H. Wang, Z. Li, A. Kohandehghan, K. Cui, Z. Xu, B. Zahiri, X. Tan, E. M. Lotfabad, B. C. Olsen, D. Mitlin, *ACS Nano* **2013**, *7*, 11004-11015.

[5] Z. Zhu, F. Cheng, J. Chen, *J. Mater. Chem. A* **2013**, *1*, 9484-9490.

[6] X.-L. Wu, Y.-G. Guo, J. Su, J.-W. Xiong, Y.-L. Zhang, L.-J. Wan, *Adv. Energy Mater.* **2013**, *3*, 1155-1160.

[7] J. S. Ko, V. V. T. Doan-Nguyen, H.-S. Kim, X. Petrissans, R. H. DeBlock, C. S. Choi, J. W. Long, B. S. Dunn, *J. Mater. Chem. A* **2017**, *5*, 18707-18715.
